# Mitochondrial DNA Haplogroup Background Affects LHON, but Not Suspected LHON, in Chinese Patients

**DOI:** 10.1371/journal.pone.0027750

**Published:** 2011-11-15

**Authors:** A-Mei Zhang, Xiaoyun Jia, Rui Bi, Antonio Salas, Shiqiang Li, Xueshan Xiao, Panfeng Wang, Xiangming Guo, Qing-Peng Kong, Qingjiong Zhang, Yong-Gang Yao

**Affiliations:** 1 Key Laboratory of Animal Models and Human Disease Mechanisms of the Chinese Academy of Sciences & Yunnan Province, Kunming Institute of Zoology, Kunming, China; 2 State Key Laboratory of Ophthalmology, Zhongshan Ophthalmic Center, Sun Yat-sen University, Guangzhou, China; 3 Unidade de Xenética, Instituto de Medicina Legal and Departamento de Anatomía Patolóxica e Ciencias Forenses, Facultade de Medicina, Universidade de Santiago de Compostela, Galicia, Spain; 4 State Key Laboratory of Genetic Resources and Evolution, Kunming Institute of Zoology, Chinese Academy of Sciences, Kunming, China; 5 Graduate School of the Chinese Academy of Sciences, Beijing, China; University of Florida, United States of America

## Abstract

Recent studies have shown that mtDNA background could affect the clinical expression of Leber hereditary optic neuropathy (LHON). We analyzed the mitochondrial DNA (mtDNA) variation of 304 Chinese patients with m.11778G>A (sample #1) and of 843 suspected LHON patients who lack the three primary mutations (sample #2) to discern mtDNA haplogroup effect on disease onset. Haplogroup frequencies in the patient group was compared to frequencies in the general Han Chinese population (*n* = 1,689; sample #3). The overall matrilineal composition of the suspected LHON population resembles that of the general Han Chinese population, suggesting no association with mtDNA haplogroup. In contrast, analysis of these LHON patients confirms mtDNA haplogroup effect on LHON. Specifically, the LHON sample significantly differs from the general Han Chinese and suspected LHON populations by harboring an extremely lower frequency of haplogroup R9, in particular of its main sub-haplogroup F (#1 vs. #3, *P-value* = 1.46×10^−17^, OR = 0.051, 95% CI: 0.016–0.162; #1 vs. #2, *P-value* = 4.44×10^−17^, OR = 0.049, 95% CI: 0.015–0.154; in both cases, adjusted *P*-value <10^−5^) and higher frequencies of M7b (#1 vs. #3, adjusted *P*-value = 0.001 and #1 vs. #2, adjusted *P*-value = 0.004). Our result shows that mtDNA background affects LHON in Chinese patients with m.11778G>A but not suspected LHON. Haplogroup F has a protective effect against LHON, while M7b is a risk factor.

## Introduction

The three primary mutations (m.3460G>A in the *MT-ND1* gene, m.11778G>A in the *MT-ND4* gene, and m.14484T>C in the *MT-ND6* gene) on mitochondrial DNA (mtDNA) have been identified to be the essential factors for Leber hereditary optic neuropathy (LHON, OMIM 535000). It is estimated that over 95% of LHON patients have one of the three primary mutations [1]–[4]. Despite decades of extensive study on LHON, several enigmas remain unresolved. Previous studies suggested that incomplete penetrance and gender bias in the clinical expression of LHON were modulated by many factors, such as mtDNA background, nuclear genes and environmental factors [5]–[11].

By way of replicating the same findings in different and independent cohorts, it has been well established in European LHON patients that haplogroups J2, J1 and K increase the risk of blindness in patients with m.11778G>A, m.14484T>C and m.3460G>A, respectively, and haplogroup H is suggested to be a protective factor for LHON patients with m.11778G>A [5], [12]–[14]. Our previous study of Chinese families with m.11778G>A showed that haplogroup M7b1′2 could increase the risk of vision loss but haplogroup M8a might produce a protective effect [15]. However, frequencies of haplogroups M7b1′2 and M8a are not significantly altered in LHON pedigrees compared to the normal controls despite their apparent background effect on the penetrance [15]. This pattern is quite different from that of European patients, in which there is an internal consistency of the haplogroup association, namely, haplogroup J is present at an increased frequency in LHON families with m.11778G>A and m.14484T>C, and subdivisions of this haplogroup show increased penetrance [5]. Dissecting haplogroups in European LHON families have shown that the increased frequency of J was indeed due to an increased frequency of two specific nested sub-clades of J (one deriving from J1 and another deriving from J2) [5], [14]. Insufficient sampling (limited statistical power), and/or potential population stratification may account for the lack of internal consistency in our observation [15].

Besides the LHON patients with the primary mutations, there are many suspected LHON patients expressing LHON clinical features but carrying none of the three primary mutations [16]. The exact pathogenic mechanism of these heterogeneous patients with suspected LHON has not been well examined. Rare pathogenic mutations, e.g. m.3635G>A, might account for the disease expression in a very small portion of these patients (Ref. [17], [18] and references therein). The *MT-ND1* and *MT-ND6* genes were found to be mutational hotspots for LHON patients without the three primary mutations in European populations [19], [20]. Similarly, we found that the *MT-ND1* and *MT-ND5* genes are mutational hotspots in Chinese suspected LHON patients with a family history but lacking the three primary mutations [21]. Whether there is an effect of mtDNA background on suspected LHON has not been tested so far.

In order to further verify the mtDNA haplogroup effect on LHON and to discern the potential effect of mtDNA background in cases of suspected LHON, we dissected the matrilineal pool of 304 LHON samples with m.11778G>A and 843 suspected LHON samples lacking the three primary mutations. We further sequenced four complete M7b mtDNA genomes and evaluated haplogroup-specific genetic variants that may be relevant for the haplogroup association with LHON. Our study provided an updated profile for the effect of mtDNA background on LHON in Chinese populations.

## Materials and Methods

### Ethics statement

Written informed consents conforming to the tenets of the Declaration of Helsinki were obtained from each participant prior to the study. The institutional review boards of the Zhongshan Ophthalmic Center and the Kunming Institute of Zoology approved this study.

### Patients

The patients received ophthalmological examinations at the Pediatric and Genetic Clinic of the Eye Hospital, Zhongshan Ophthalmic Center and/or local medical centers, and were categorized according to the expression of LHON clinical features (e.g. painless, acute or sub-acute vision deterioration without apparent reasons). LHON patients with primary mutation m.14484T>C or m.3460G>A were reported elsewhere [22], [23] and were not discussed in this study. 175 LHON patients with m.11778G>A had a family history and were described in our previous study [15]; these patients were reanalyzed with the new samples in this study. LHON patients with both m.11778G>A and m.593T>C (n = 14) and suspected LHON patients with m.593T>C (n = 12) were discussed in a separate paper [24]. Patients without the three primary mutations of LHON were simply regarded as patients with suspected LHON, despite that this grouping included a very small proportion of LHON patients that were caused by other rare primary LHON mutations. Blood samples were collected from patients in vacuum tubes containing EDTA. For some cases, blood samples were collected on filtered papers.

### Screening of primary LHON mutations, sequencing the mtDNA control region and complete genome

The PCR-single strand conformation polymorphism (PCR-SSCP) and allele specific PCR (AS-PCR) methods were jointly used to detect the three primary mutations as previously described [25]. The presence of the three primary mutations in the patients was independently screened in Kunming using method described by Bi et al. [26]. The mtDNA control region sequence of each patient was amplified by using primer pair (L15594: 5′-CGCCTACACAATTCTCCGATC-3′ and H901: 5′-ACTTGGGTTAATCGTGTGACC-3′) and the following PCR condition: one pre-denaturation cycle at 94°C for 5 min; 30 cycles at 94°C for 30 sec, 52°C for 30 sec, 72°C for 2 min; and one extension cycle at 72°C for 10 min. The PCR products were purified and sequenced by sequencing primers, which were used in our previous study [27], on a 3730 DNA analyzer (Applied Biosystems). Each sample was sequenced for an approximate 1.4 kb fragment (mtDNA regions 16033–16569 and 1–850; numbering according to the revised Cambridge reference sequence (rCRS) [28]) which cover the mtDNA control region, the *MT-TF* gene and part of the *MT-RNR1* gene. We determined the entire mtDNA sequences for four LHON patients (Fig. S1) belonging to haplogroup M7b1′2 using the same procedure and condition described in our previous study [27]. Novelty and potential pathogenicity for those non-synonymous private variants in these lineages were analyzed using the same method reported by Bandelt et al. [29].

### mtDNA haplogroup classification and statistical analysis

The sequences were compared with the rCRS [28] and the variants in each mtDNA sequence were recorded and were further checked by using the MitoTool (www.mitotool.org) [30]. We classified each sample based on the haplogroup motifs that were identified according to the PhyloTree (http://www.phylotree.org; mtDNA tree Build 9, 20 Jun 2010) [31]. For samples which could not be unambiguously determined by the mtDNA control region sequence variants, PCR- restriction fragment length polymorphism (RFLP) analysis for specific coding region variant(s) was performed, e.g. a check of −5176*Alu*I was performed to confirm haplogroup D status.

We first compared the matrilineal composition of suspected LHON patients (*n* = 843) to 1,689 pooled Han Chinese mtDNAs from the general populations (Table S1), to discern the potential effect of mtDNA background on the onset of suspected LHON. Under the null hypothesis that the suspected LHON patients had a similar matrilineal composition as the pooled Han Chinese, the suspected LHON patients could be used as a second control group for comparison to the LHON patients. We then compared the newly obtained 304 LHON patients with m.11778G>A to the suspected LHON samples and the pooled Han Chinese samples, respectively, to discern the mtDNA haplogroup background effect on LHON and to verify the pattern as observed in our previous studies based on limited sample sizes [15]. We next pooled the newly obtained LHON patients (*n* = 304) with the reported LHON patients (*n* = 175; [15]). Combining these two different datasets provide more statistical power than the strategy of analyzing both data sets separately (Methods S1) [32].

Pearson's chi-square test with a one degree of freedom was used to assess the significance of the differences observed in haplogroup frequencies between cases and controls. The Fisher Exact test (two tailed) was applied to those cases with cell counts below five. A permutation test was used to address the issue of multiple testing in haplogroup association test; 100,000 permutations were used in all comparisons. The statistical package SPSS (version 15.0) and Stata v.8 (http://www.stata.com) were used for all the computations; adjusted *P*-values below 0.05 were considered to be statistically significant.

## Results

### Patient features

A total of 1,322 unrelated subjects collected from different provinces in China were enrolled in the current analysis based on the availability of DNA after the initial primary mutation screening. These patients included 479 LHON patients with mutation m.11778G>A (including 175 samples reported in our previous study [15] and 304 samples newly sequenced) and 843 suspected LHON patients who expressed clinical features but did not harbor any of the three primary mutations. Only one out of 479 patients had the heteroplasmic mutation m.11778G>A. The overall frequency of patients with heteroplasmic mutation m.11778G>A was thus very low (0.21%; 1/479) in these Chinese patients based on the limitation of detection sensitivity of our approach. The mtDNA control region sequence variations for each patient are listed in the online Tables S2 and S3, and the sequences can be retrieved from GenBank under the Accession Numbers HM632037–HM632340 (for 304 LHON patients with m.11778G>A) and HM632341–HM633183 (for 843 suspected LHON patients).

### mtDNA Haplogroup distribution in patients with suspected LHON

**Figure 1:**
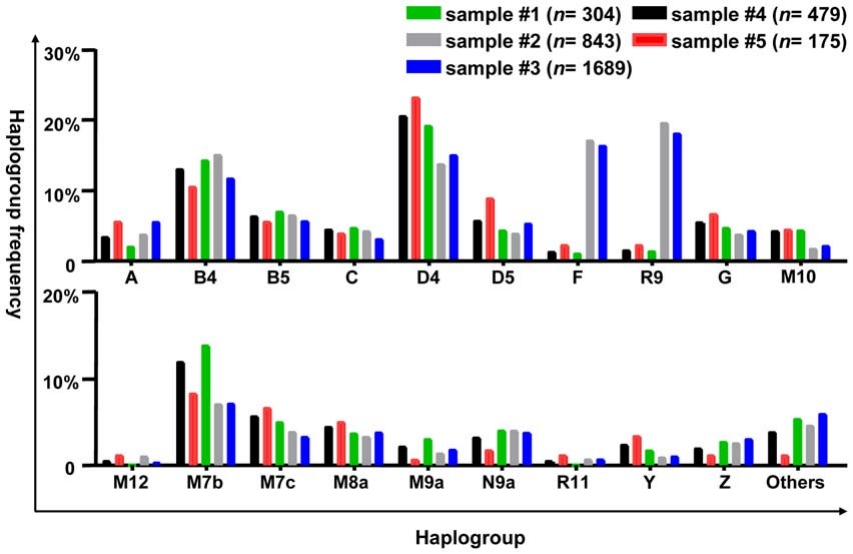
mtDNA haplogroup distribution frequency of different cohorts of LHON patients with m.11778G>A and control samples. Detailed information for the newly sequenced LHON patients with m.11778G>A (sample #1, *n* = 304), reported LHON patients with m.11778G>A in our previous study (sample #5, n = 175; Ref. [15]), pooled LHON patients (sample #4, n = 479), suspected LHON patients (sample #2, *n* = 843) and the reported Han Chinese from the general populations (sample 3, *n* = 1,689; Table S1) are listed in Tables 2 and 3 and Tables S4, S5, S6, S7. Haplogroup R9 contains samples belonging to haplogroup F and its subhaplogroups. We lumped together all these haplogroups that occurred in less than five individuals per haplogroup as others.

We first dissected the matrilineal genetic components in the suspected LHON (sample #2) and compared to the pooled Han Chinese from the general populations (sample #3, *n* = 1,689). As shown in Table 1 and Fig. 1, the overall matrilineal composition of the 843 suspected LHON patients resembled that of the pooled Han Chinese. There was no statistically significant difference for any of the haplogroup classes between the two populations. Therefore, the suspected LHON sample, despite of its miscellaneous nature of the disease, could be used as the second independent control sample to compare with LHON patients with m.11778G>A in the subsequent analyses.

**Table 1:** Haplogroup frequencies and Pearson's chi-square test in 843 patients with suspected LHON and 1,689 Han Chinese from the general populations.

Haplogroup	Suspected LHON	Pooled Han Chinese^a^	*P*-value^b^	Adjusted*P*-value^c^	OR	95% CI
A	31	92	0.064	0.686	0.663	0.437–1.004
B4	126	196	0.021	0.314	1.339	1.052–1.703
B5	54	94	0.448	1.000	1.161	0.822–1.640
C	35	51	0.172	0.969	1.391	0.897–2.157
D4	115	252	0.423	1.000	0.901	0.710–1.143
D5	32	88	0.139	0.934	0.718	0.475–1.085
R9^d^	164	305	0.394	0.999	1.413	0.804–2.483
F	143	274	0.677	1.000	1.055	0.845–1.317
F1	99	193	0.229	0.991	1.175	0.903–1.530
F1a	71	132	0.281	0.997	1.165	0.882–1.541
F2	17	48	0.216	0.988	1.421	0.812–2.487
F2a	16	22	0.246	0.994	1.466	0.766–2.806
F3	12	15	0.216	0.988	1.612	0.751–3.458
F3a	12	13	0.117	0.887	1.862	0.846–4.098
F4	7	8	0.270	0.996	1.759	0.636–4.868
G	31	71	0.598	1.000	0.870	0.566–1.338
M10	14	35	0.579	1.000	0.798	0.427–1.492
M12	8	4	0.031	0.436	4.036	1.212–13.441
M7b	59	119	1.000	1.000	0.993	0.718–1.372
M7c	32	54	0.504	1.000	1.195	0.765–1.865
M8a	27	63	0.575	1.000	0.854	0.540–1.351
M9a	11	29	0.539	1.000	0.757	0.376–1.522
N9a	33	62	0.847	1.000	1.069	0.695–1.645
R11	5	10	1.000	1.000	1.002	0.341–2.940
Y	7	16	0.944	1.000	0.876	0.359–2.136
Z	21	50	0.585	1.000	0.837	0.500–1.404

a Pooled Han Chinese were from reported populations (see Table S1 for more information).

b Two tailed Fisher exact test was applied instead of a Pearson chi-square test in cases containing cell counts below five.

c Adjusted *P*-value: adjustment of *P*-values was carried out with a permutation-based approach; number of permutations = 100,000; OR (95% CI): Odds Ratio (95% Confidence Interval).

d Note that haplogroup F is a sub-haplogroup of haplogroup R9 and therefore the number of F mtDNAs are also included here.

### mtDNA Haplogroup distribution in LHON patients with m.11778G>A

To compare with the pattern that was reported in our previous studies based on limited number of patients [15], we treated the newly obtained 304 LHON patients as an independent sample (sample #1) and compared to the two control population groups. We found that haplogroups R9 (including its subhaplogroups F, F1, F1a) and M7b presented remarkable differences between the LHON patients and the controls after adjusting for multiple test (Tables 2 and 3). The LHON sample significantly differs from the general Han Chinese and suspected LHON populations by harboring an extremely lower frequency of haplogroup R9 (#1 vs. #3, *P-value* = 7.62×10^−19^, OR = 0.061, 95% CI: 0.022–0.164; #1 vs. #2, *P-value* = 5.19×10^−19^, OR = 0.055, 95% CI: 0.020–0.150; in both cases, adjusted *P*-value <10^−5^), in particular of its main sub-haplogroup F (#1 vs. #3, *P-value* = 1.46×10^−17^, OR = 0.051, 95% CI: 0.016–0.162; #1 vs. #2, *P-value* = 4.32×10^−17^, OR = 0.049, 95% CI: 0.015–0.154; in both cases, adjusted *P*-value <10^−5^) and higher frequencies of M7b (#1 vs. #3, *P-value* = 7.00×10^−5^, adjusted *P*-value = 0.001 and #1 vs. #2, *P-value* = 3.23×10^−4^, adjusted *P*-value = 0.004). Distribution of the other haplogroups had no statistical difference between the two comparisons after adjustment of *P*-values.

**Table 2:** Haplogroup frequencies and Pearson's chi-square test in 304 LHON patients with m.11778G>A and 843 patients with suspected LHON.

Haplogroup	LHON	Suspected LHON	*P*-value^a^	Adjusted *P*-value^b^	OR	95% CI
A	6	31	0.150	0.931	0.527	0.218–1.277
B4	43	126	0.735	1.000	0.938	0.645–1.363
B5	21	54	0.761	1.000	1.084	0.643–1.827
C	14	35	0.738	1.000	1.114	0.591–2.101
D4	58	115	0.023	0.304	1.493	1.055–2.112
D5	13	32	0.712	1.000	1.132	0.586–2.187
R9^c^	4	164	5.190×10^−19^	<10^−5^	0.055	0.020–0.150
F	3	143	4.319×10^−17^	<10^−5^	0.049	0.015–0.154
F1	2	99	1.003×10^−11^	<10^−5^	0.050	0.012–0.203
F1a	1	71	6.614×10^−9^	<10^−5^	0.036	0.005–0.259
F2	0	17	9.725×10^−3^	0.141	0.731	0.706–0.757
F3	1	12	0.203	0.979	0.229	0.030–1.765
F3a	1	12	0.203	0.979	0.229	0.030–1.765
F4	0	7	0.200	0.977	0.733	0.708–0.759
G	14	31	0.475	1.000	1.265	0.663–2.411
M10	13	14	0.010	0.144	2.645	1.229–5.694
M12	0	8	0.119	0.872	0.733	0.708–0.759
M7b	42	59	3.233×10^−4^	0.004	2.130	1.400–3.241
M7c	15	32	0.391	0.999	1.315	0.702–2.465
M8a	11	27	0.729	1.000	1.135	0.556–2.316
M9a	9	11	0.059	0.631	2.308	0.947–5.624
N9a	12	33	0.980	1.000	1.009	0.514–1.979
R11	0	5	0.333	0.999	0.734	0.709–0.760
Y	5	7	0.232	0.989	1.997	0.629–6.340
Z	8	21	0.894	1.000	1.058	0.464–2.414

a Two tailed Fisher exact test was applied instead a Pearson chi-square test in cases containing cell counts below five.

b Adjusted *P*-value: adjustment of *P*-values was carried out with a permutation-based approach; number of permutations = 100,000; OR (95% CI): Odds Ratio (95% Confidence Interval).

c Note that haplogroup F is a sub-haplogroup of haplogroup R9 and the number of F mtDNAs are also included here.

**Table 3:** Haplogroup frequencies and Pearson's chi-square test in 304 LHON patients with m.11778G>A and 1,689 Han Chinese from general populations.

Haplogroup	LHON	Pooled Han Chinese^a^	*P-*value^b^	Adjusted *P*-value^c^	OR	95% CI
A	6	92	0.010	0.144	0.350	0.152–0.806
B4	43	196	0.210	0.981	1.255	0.880–1.790
B5	21	94	0.355	1.000	1.259	0.772–2.055
C	14	51	0.152	0.948	1.551	0.847–2.838
D4	58	252	0.065	0.673	1.344	0.980–1.844
D5	13	88	0.494	1.000	0.813	0.448–1.474
R9^d^	4	304	7.621×10^−19^	<10^−5^	0.061	0.022–0.164
F	3	274	1.461×10^−17^	<10^−5^	0.051	0.016–0.162
F1	2	193	2.822×10^−12^	<10^−5^	0.051	0.013–0.208
F1a	1	132	5.356×10^−9^	<10^−5^	0.039	0.005–0.280
F2	0	48	7.125×10^−4^	0.012	0.844	0.828–0.860
F3	1	15	0.492	1.000	0.368	0.049–2.799
F3a	1	13	0.708	1.000	0.425	0.055–3.265
F4	0	8	0.616	1.000	0.847	0.831–0.863
G	14	71	0.750	1.000	1.100	0.612–1.978
M10	13	35	0.021	0.288	2.111	1.104–4.039
M12	0	4	1.000	1.000	0.847	0.831–0.863
M7b	42	119	6.669×10^−5^	0.001	2.115	1.453–3.078
M7c	15	54	0.127	0.908	1.572	0.875–2.822
M8a	11	63	0.925	1.000	0.969	0.505–1.861
M9a	9	29	0.144	0.936	1.746	0.818–3.727
N9a	12	62	0.814	1.000	1.078	0.574–2.026
R11	0	10	0.376	1.000	0.847	0.831–0.863
Y	5	16	0.273	0.995	1.749	0.636–4.809
Z	8	50	0.754	1.000	0.886	0.416–1.888

a Pooled Han Chinese were from reported populations (see Table S1 for more information).

b Two tailed Fisher exact test was applied instead a Pearson chi-square test in cases containing cell counts below five.

c Adjusted *P*-value: adjustment of *P*-values was carried out with a permutation-based approach; number of permutations = 100,000; OR (95% CI): Odds Ratio (95% Confidence Interval).

d Note that haplogroup F is a sub-haplogroup of haplogroup R9 and the number of F mtDNAs are also included here.

We further pooled the newly analyzed LHON patients with the previously reported LHON patients [15] as one population for comparison, to increase the power of the statistical test [32]. The dissection of matrilineal genetic components in the enlarged LHON population [479 LHON patients with m.11778G>A (sample #4)] and comparison to the two control groups [suspected LHON (sample #2) and pooled Han Chinese from the general populations (sample #3)] revealed several interesting yet important features (Fig. 1 and Tables S4 and S5). First, haplogroups M7b (#4 vs. #3, adjusted *P*-value = 0.018; #4 vs. #2, adjusted *P-*value = 0.047) and possibly D4 (#4 vs. #3, adjusted *P-*value = 0.085; #4 vs. #2, adjusted *P-*value = 0.031) are both more prevalent in patients with LHON than in the general Han Chinese group. Most of LHON patients with m.11778G>A belonging to haplogroup M7b could be further classified into M7b1′2 (77.19%, 44/57). Second, haplogroup R9, and more specifically, its sub-haplogroup F has a strikingly lower frequency in LHON patients with m.11778G>A than in the other two control populations (#4 vs. #3, nominal *P-value* = 1.999×10^−24^, adjusted *P*-value <10^−5^; #4 vs. #2, nominal *P-value* = 9.133×10^−23^, adjusted *P*-value <10^−5^). This significance is still strongly maintained for several F sub-haplogroups (F, F1, F1a), as also indicated when analyzing the newly sequenced LHON patients (Tables 2 and 3) and the reported LHON patients with m.11778G>A in our previous study (Ref. [15] and references therein) (Tables S6 and S7), separately.

### Complete mtDNA tree for LHON patients belonging to haplogroup M7b1′2

**Figure 2:**
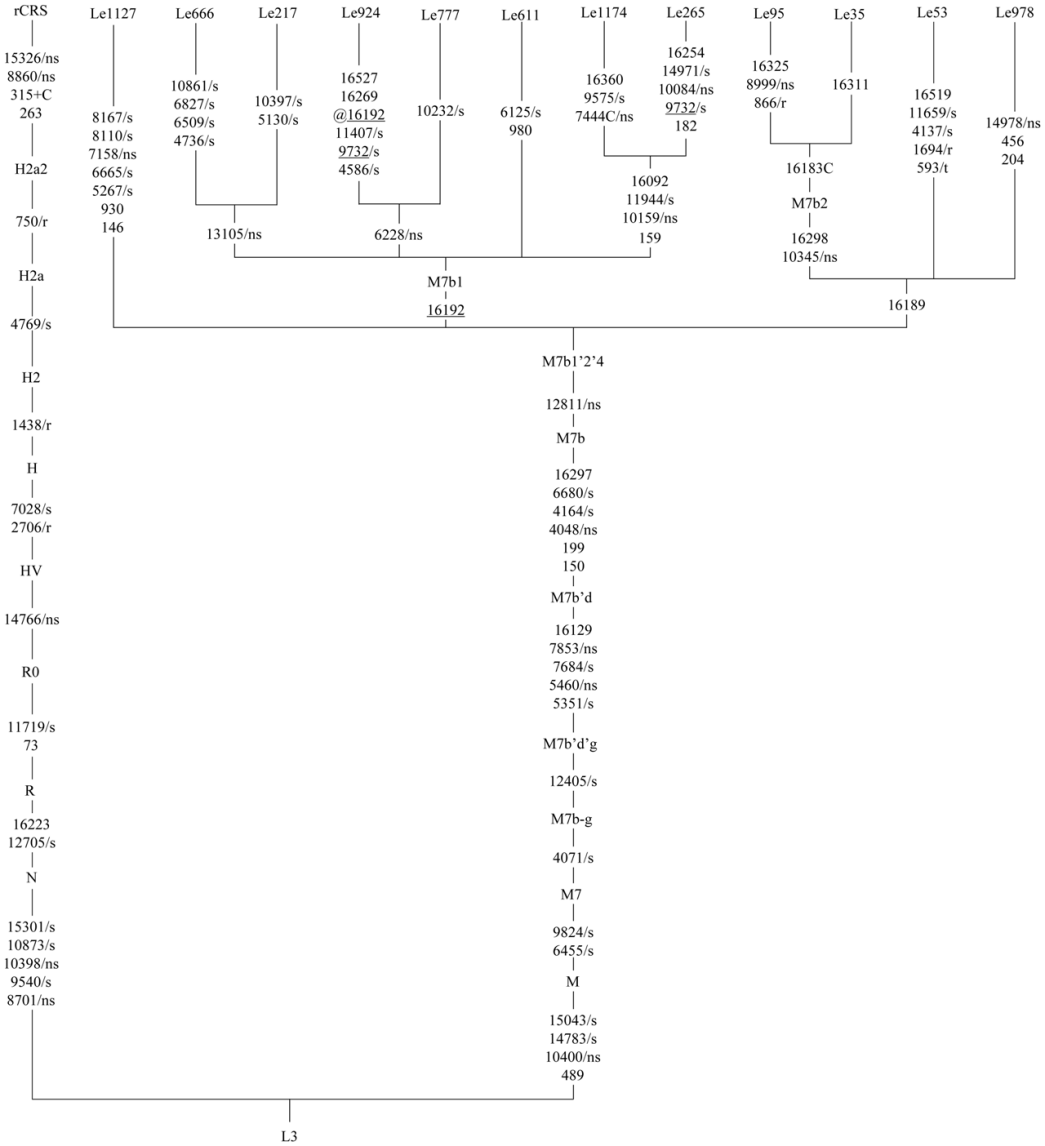
Classification tree of M7b complete mtDNA sequences, plus the revised Cambridge reference sequence (rCRS) [**28**]. Five Chinese LHON mtDNAs (including Le53 (GenBank accession number JF896798) which was reported in our recent study [24]) belonged to haplogroup M7b1′2 were analyzed. The length polymorphisms of the C-tracts in region 303–309 were not considered. Mutations on each uninterrupted branch segment are listed in an arbitrary order. Recurrent mutations are underlined. The synonymous and non-synonymous coding-region variants in each mtDNA are denoted by “/s” and “/ns”, respectively. Variants in the rRNA genes and tRNA genes are denoted by “/r” and “/t”, respectively.

In order to further evaluate the effect of the haplogroup-specific variants that account for the haplogroup association with LHON, we determined four M7b complete mtDNAs (GenBank accession numbers JN645818–JN645821), and analyzed these sequences with those from published sources [24]. Five private variants (including four non-synonymous and one rRNA variant) were found in the four patients, but none of these variants (with the exception of m.8999T>C [p.V158A]) were conserved according to the evolutionary analysis performed by the MitoTool web server (Table 4). Two of these private variants (m.13105A>G and m.14978A>G) were reported as haplogroup-specific variants for other haplogroups. Interestingly, m.6228C>T (p.L109F) in patient Le777 and m.13105A>G (p.I257V) in patient Le666 were previously reported in LHON patients belonging to haplogroup M7b1′2 [15]. We presented sequence variants in each mtDNA in a classification tree following our previous strategy [15] (Fig. 2). The newly determined M7b sequences indicated two novel clades within M7b1, which were defined by m.13105A>G and m.6228C>T, respectively. We counted the number of private variants that were located in the terminal branches of the phylogenetic tree in each of the 13 mitochondrial DNA encoding genes in 12 LHON patients sequenced in this study and our previous study [15], and compared to that of 69 reported complete mtDNAs from the general populations (refer to [21] and references therein). There was no difference regarding the occurrence of private non-synonymous (NS) and synonymous (S) substitutions between the LHON patients and controls from the general populations; note that the sample size was very limited and this result should be treated with caution (Table S8).

**Table 4:** Private non-synonymous and mt-rRNA variants in four Chinese LHON patients with m.11778G>A and a haplogroup status of M7b1′2.

Sample	Haplogroup	Nucleotide variant (Amino acid change)	Gene	ConservationIndex^a^	Reported^b^(population context)	Reported^b^(disease context)	Haplogroup specific variant^c^
Le95	M7b2	m.8999T>C (p.V158A)	*MT-ATP6*	1.000	Yes	No	No
		m.866A>G	*MT-RNR1*	0.256	Yes	No	No
Le666	M7b1	m.13105A>G (p.I257V)	*MT-ND5*	0.256	Yes	Yes	L5c, L3b, L3e2a, etc.
Le777	M7b1	m.6228C>T (p.L109F)	*MT-CO1*	0.140	Yes	Yes	No
Le978	M7b1′2′4	m.A14978G (p.I78V)	*MT-CYB*	0.651	Yes	Yes	C7a1

a The evolutionary conservation analysis was performed by comparing human mtDNA (GenBank accession no. J01415) to 43 different primate species by using the MitoTool (http://www.mitotool.org) [30]. A conservation index of 1 for certain variant means that this position is conserved in all species considered for comparison.

b Web and dataset based searches were performed on September 5, 2011, following the same strategy described in our previous study [29] (e.g. both ‘G6249A mtDNA’ and ‘m.6249G>A mtDNA’ were queried).

c The column “Haplogroup specific variant” refers to the presence of the corresponding variant in the world mtDNA phylogenetic tree displayed at http://www.phylotree.org/tree/main.htm (mtDNA tree Build 12; 20 Jul 2011) [31]. We listed the haplogroup name if it was characterized by the private variants identified in our newly generated M7b sequences.

## Discussion

In recent years, many studies reported that mtDNA background affects the expression of human disorders [33]–[37], although some positive findings could respond to false positives [38]. Among these studies, LHON is probably the most extensively studied disease that has been reported to be modulated by mtDNA haplogroups [5], [12]–[15]. In this study, we analyzed 479 unrelated LHON samples with m.11778G>A (including 175 LHON patients reported in our previous study [15] and 304 patients newly sequenced) and 843 suspected LHON patients without the three primary mutations, with the aim of primarily addressing the following questions: (1) does mtDNA haplogroup background contribute to the suspected LHON? (2) will the enlarged LHON patients with m.11778G>A discern the internal consistency of the haplogroup association of LHON penetrance? Dissecting the matrilineal components of the enlarged sample size of LHON patients with m.11778G>A and the suspected LHON patients did provide new insightful information to both questions.

In contrast to the pattern that was observed in the LHON patients with m.11778G>A [15], the overall matrilineal composition of the suspected LHON patients resembles that of the pooled Han Chinese, showing no evident effect from mtDNA background (Fig. 1 and Tables 1, 2, 3). This result was not surprising, as the suspected LHON cohort was quite heterogeneous and might be caused by nuclear gene defects, as well as, some rare mtDNA pathogenic mutations. Indeed, we analyzed some of these patients and identified mutations in the *OPA1* gene, suggesting that these patients should be classified as autosomal dominant optic atrophy (ADOA; authors' unpublished data). Nonetheless, the suspected LHON population could be used as a suitable control population for the LHON patients carrying m.11778G>A. In addition, both LHON carriers and none carriers of m.11778G>A were collected in the same geographic origin, a fact that helps to prevent the undesirable effects of population stratification in inflating type I error.

A direct comparison of the frequencies of the matrilineal components between the LHON population and the suspected LHON population or between the LHON population and the pooled Han Chinese from the general populations yields meaningful information on the mtDNA background effect on LHON. Haplogroup M7b1′2, which was found to increase vision loss risk in the presence of m.11778G>A [15], had a significantly higher occurrence in LHON patients with this mutation than in patients with suspected LHON or in Han Chinese from the general populations (Tables 2 and 3 and Tables S4 and S5). This observation provides direct evidence for the internal consistency of association of haplogroup M7b1′2 with clinical expressions of LHON, as predicted by our previous study [15]. Note that in our previous study [15], the sample size for the control Han Chinese population was relatively small (n = 408) and this would bias the comparison for certain haplogroup. This defect had been corrected by including more Han Chinese samples from the general populations (n = 1,689) in the current analysis (Tables S6 and S7). As shown in Tables S2 and S3, most of the mtDNAs belonging to M7 and its subhaplogroups carry different control region profiles, indicating no close maternal relationship between these carriers; the different frequencies observed between cohorts are unlikely to be caused by close kinship. Analysis of the complete mtDNA sequence variation in those LHON patients belonging to M7b further supported our previous result that variant m.12811T>C would account for the haplogroup association with LHON [15]. It seems that no other private variants would account for the overall increased risk for visual loss for pedigrees belonging to haplogroup M7b1′2.

On the other hand, haplogroup M8a was found to reduce the clinical penetrance of mutation m.11778G>A in Chinese families in our previous study [15]. However, even with increased sample size of LHON patients, there was no indication, as it was expected, of a reduced frequency of M8a in LHON patients relative to control populations.

Consistent with the previous observations [15], haplogroup D4 has a substantially higher frequency in the LHON patients (for 304 new patients, 58/304 = 19.08%; for pooled 479 patients, 98/479 = 20.50%) than in patients with suspected LHON (13.64%, 115/843) or in the pooled Han Chinese population (14.92%, 252/1689), though the adjusted *P*-value is not statistically significant (but marginal for pooled samples) between LHON patients and pooled Han Chinese. Because more than half of the LHON patients analyzed in this study were sporadic cases and we lacked the detailed clinical information for some families with self-reported disease history, we were unable to perform a correlation analysis to discern the effect of haplogroup D4 on the clinical expression of LHON. We failed to discern such an effect in our previous analysis for LHON families with m.11778G>A (Ref. [15] and references therein). Haplogroup D4 is defined by a string of non-synonymous variants (m.5178C>A in the *MT-ND2* gene and m.8414C>T in the *MT-ATP6* gene), synonymous variants (m.4883C>T in the *MT-ND2* gene, m.14668C>T in the *MT-ND6* gene) and one variant (m.3010G>A) in the *MT-RNR2* gene. It is most likely that the non-synonymous variants of haplogroup D4 might be responsible for the potentially functional effect. Indeed, variant m.5178C>A had been reported to be associated with a variety of human disorders [39] and longevity [40], despite the fact that there was controversy regarding its role in longevity [41], and the functional assay did not support the notion that this variant could alter mitochondrial matrix pH and intracellular calcium dynamics [42]. The exact role of haplogroup D4 in LHON needs further study.

Haplogroup F was very rare in the LHON patients (for 304 new patients, 3/304 = 0.99%; for pooled 479 patients, 6/479 = 1.25%), compared with its occurrence in the pooled Han Chinese from the general populations (274/1689 = 16.22%) and in the suspected LHON patients (143/843 = 16.96%). This result again confirmed the observation in our previous study [15] and a recent study in LHON patients from Thailand [10], in which an extremely low frequency of haplogroup F (including its subhaplogroups) was observed in LHON patients with m.11778G>A. Although the confounding effect of population stratification cannot be fully disregarded, the clear-cut different frequency of haplogroup F in two independent Chinese LHON patient cohorts (patients newly determined in this study and reported in our previous study [15]; Tables 2 and 3, Tables S6 and S7) with respect to the two independent control groups (suspected LHON patients plus the general Han Chinese) is so pronounced that it is reasonable to consider an alternative hypothesis that would involve haplogroup F as a protective factor in LHON patients. Because only six patients with m.11778G>A belong to haplogroup F in the LHON patients and half of them were sporadic, we could not estimate its potential effect on the clinical expression of LHON and analysis of the complete mtDNA sequence of some of these subjects yielded no insightful information [15]. Intriguingly, we also noticed a very low prevalence of F lineages (1.92%; 1/52) in LHON subjects with m.14484T>C in our recent study [23]. The exact reason for the extremely low frequency of F lineages in LHON patients with m.11778G>A or m.14484T>C remains enigmatic. Notably, haplogroup F is one of the sub-clades of haplogroup R9, and R9 showed evidently lower frequency in LHON patients than in the two control samples (Tables 2 and 3, Tables S4, S5, S6, S7), so the ancient genetic variants of haplogroup R9 might contribute to the pathogenesis of LHON patients. Variant m.13928G>C was the only non-synonymous nucleotide change at the basal branch level of haplogroup R9 and caused serine to threonine at position 531 of the MT-ND5 protein, but this variant did not alter the hydrophobicity significantly and occurred multiple times in different populations and haplogroup backgrounds (cf. www.phylotree.org). Similar to mtDNA variant m.12811T>C which has been considered as the cause for the increased risk of M7b1′2 [15] and occurred in worldwide regions, the conservation and the hydrophobicity of m.13928G>C did not prove itself to be the possible pathogenic mutation, although recent studies showed that ancient variation may influence mtDNA replication and transcription [43] or contribute to cellular physiological changes in the presence of the primary mutations [44]. The deletion of adenine at position 249 is a characteristic variant of haplogroup F and other Asian haplogroups CZ, M31a1 and M36d1 [31]. Variant m.249delA was located in the mtTF1 binding site (MT-TFX or TFAM) which binds with mtTF and controls mtDNA transcription [45]. Some single base changes within this region have been identified to alter protein binding efficiency [43]. Whether m.249delA could affect the efficiency of mtDNA transcription remains unclear.

Population stratification could in part explain the results obtained in the present study. Monitoring population stratification is complex in mtDNA studies, but the use of two control groups, as performed in this study, helps to prevent its undesirable consequences in case-control studies. On the other hand, whether treating the newly analyzed 304 LHON patients with m.11778G>A and the reported LHON patients in our previous study [15] as independent samples, or pooled these new patients with the previously reported LHON patients [15] as an enlarged sample, we could get consistent findings for a lower frequency of the nested haplogroups R9, F, F1, and F1a. Therefore, we believe that different distribution frequencies of these haplogroups in LHON patients and control populations were biologically meaningful and might be shaped by the selection effect during the past.

One limitation of the current study is that we lacked detailed clinical information for these patients newly sequenced in this study (most of these patients were collected more than five years ago and there was no follow-up information), we could not perform a systematic estimation for LHON penetrance, disease severity and propensity to spontaneous recovery of visual acuity, as well as, their correlation with mtDNA genetic background. In future study, we will attempt to collect all detailed clinical data for each newly recruited patient and fulfill the task.

In summary, analysis of more newly collected LHON patients with m.11778G>A further supports the pattern that was observed in our previous studies [15], in that a significantly higher prevalence of haplogroups M7b and a strikingly low occurrence of haplogroup F have been confirmed. The higher frequency of M7b in patients with m.11778G>A than in control samples provides evidence for the internal consistency of association of haplogroup M7b1′2 with clinical expression of LHON. There was no evident effect of mtDNA background on Chinese patients with suspected LHON. Future studies could assist in providing further support to the present findings and provide new clues on the molecular mechanism by which a particular mtDNA background could influence clinical expression of LHON.

## Supporting Information

Figure S1 **Pedigree information for three Chinese LHON families with m.11778G>A that were sequenced for the entire mtDNA sequence.** Patient Le95 was regarded as sporadic according to our definition for sporadic patient in our recent study [23]. Affected individuals are marked by filled symbols. The proband that was sequenced for the entire mtDNA sequence in each family was marked by an arrow. The probands in these families had been analyzed for the mtDNA control region sequence variation in our previous study [15].
(DOC)

Table S1 **Information for the reported Han Chinese from the general populations.**
(DOC)

Table S2 **mtDNA sequence variation and haplogroup classification of 304 patients with LHON and m.11778G>A.**
(XLS)

Table S3 **mtDNA sequence variation and haplogroup classification of 843 patients with suspected LHON.**
(XLS)

Table S4 **Haplogroup frequencies and Pearson's chi-square test in 479 LHON patients with m.11778G>A and 843 patients with suspected LHON.**
(DOC)

Table S5 **Haplogroup frequencies and Pearson's chi-square test in 479 LHON patients with m.11778G>A and 1,689 Han Chinese from the general populations.**
(DOC)

Table S6 **Haplogroup frequencies and Pearson's chi-square test in 175 LHON patients with m.11778G>A and 843 patients with suspected LHON.**
(DOC)

Table S7 **Haplogroup frequencies and Pearson's chi-square test in 175 LHON patients with m.11778G>A and 1,689 Han Chinese from general populations.**
(DOC)

Table S8 **Comparison of the non-synonymous (NS) and synonymous (S) substitutions at the terminal branch level in the phylogenetic tree between 12 LHON patients belonging to haplogroup M7b1′2 and 69 reported Chinese complete mtDNAs from the general populations.**
(DOC)

Methods S1 **Supplementary methods for power calculations.**
(DOC)
