# Supplementary material for: Mitochondrial DNA Haplogroup Background Affects LHON, but Not Suspected LHON, in Chinese Patients
Source: PLoS One. 2011 Nov 15;6(11):e27750. doi: 10.1371/journal.pone.0027750 (PMC3216987; doi:10.1371/journal.pone.0027750)
Supplement: Table S1 — Information for the reported Han Chinese from the general populations. (DOC) [file pone.0027750.s002.doc]

Table S1. Information for the reported Han Chinese from the general populations

| Location | No. of samples | References |
| --- | --- | --- |
| Shandong | 126 | 1, 2 |
| Hubei | 42 | 2 |
| Xinjiang | 47 | 2 |
| Yunnan | 101 | 2, 4 |
| Anhui | 42 | 4 |
| Fujian | 54 | 4 |
| Gansu | 45 | 4 |
| Guangxi | 26 | 4 |
| Hunan | 16 | 4 |
| Jiangsu | 67 | 4 |
| Jiangxi | 23 | 4 |
| Liaoning | 102 | 4 |
| Neimeng | 45 | 4 |
| Qinghai | 44 | 4 |
| Sichuan | 70 | 4 |
| Shanghai | 56 | 4 |
| Shanxi | 53 | 4 |
| Zhejiang | 61 | 4 |
| Guangdong | 669 | 2, 3, 5, 6, 7 |
| Total | 1689 | — |

**Supplementary References**

1. Yao Y-G, Kong Q-P, Man X-Y, Bandelt H-J, Zhang Y-P (2003) Reconstructing the evolutionary history of China: a caveat about inferences drawn from ancient DNA. Mol Biol Evol 20: 214-219

2. Yao Y-G, Kong Q-P, Bandelt H-J, Kivisild T, Zhang Y-P (2002) Phylogeographic differentiation of mitochondrial DNA in Han Chinese. Am J Hum Genet 70: 635-651

3. Kivisild T, Tolk H-V, Parik J, Wang Y, Papiha SS, Bandelt H-J, Villems R (2002) The emerging limbs and twigs of the East Asian mtDNA tree. Mol Biol Evol 19: 1737-1751

4. Wen B, Li H, Lu D, Song X, Zhang F, He Y, Li F, Gao Y, Mao X, Zhang L, Qian J, Tan J, Jin J, Huang W, Deka R, Su B, Chakraborty R, Jin L (2004) Genetic evidence supports demic diffusion of Han culture. Nature 431: 302-305

5. Chen F, Wang S-Y, Zhang R-Z, Hu Y-H, Gao G-F, Liu Y-H, Kong Q-P (2008) Analysis of mitochondrial DNA polymorphisms in Guangdong Han Chinese. Forensic Sci Int Genet 2: 150-153

6. Wang Q, Wang P, Li S, Xiao X, Jia X, Guo X, Kong Q-P, Yao Y-G, Zhang Q (2010) Mitochondrial DNA haplogroup distribution in Chaoshanese with and without myopia. Mol Vis 16: 303-309

7. Wang W-Z, Wang C-Y, Cheng Y-T, Xu A-L, Zhu C-L, Wu S-F, Kong Q-P, Zhang Y-P (2010) Tracing the origins of Hakka and Chaoshanese by mitochondrial DNA analysis. Am J Phys Anthropol 141: 124-130
