# Supplementary material for: Mitochondrial DNA Haplogroup Background Affects LHON, but Not Suspected LHON, in Chinese Patients
Source: PLoS One. 2011 Nov 15;6(11):e27750. doi: 10.1371/journal.pone.0027750 (PMC3216987; doi:10.1371/journal.pone.0027750)
Supplement: Table S4 — Haplogroup frequencies and Pearson's chi-square test in 479 LHON patients with m.11778G>A and 843 patients with suspected LHON. (DOC) [file pone.0027750.s005.doc]

Table S4. Haplogroup frequencies and Pearson’s chi-square test in 479 LHON patients with m.11778G>A and 843 patients with suspected LHON

| Haplogroup | LHON | Suspected LHON | *P*-value ^a^ | Adjusted *P*-value ^b^ | OR | 95% CI |
| --- | --- | --- | --- | --- | --- | --- |
| A | 16 | 31 | 0.870 | 1.000 | 0.905 | 0.490-1.673 |
| B4 | 62 | 126 | 0.357 | 1.000 | 0.846 | 0.610-1.173 |
| B5 | 30 | 54 | 1.000 | 1.000 | 0.976 | 0.616-1.548 |
| C | 21 | 35 | 0.953 | 1.000 | 1.059 | 0.609-1.840 |
| D4 | 98 | 115 | 0.002 | 0.031 | 1.628 | 1.210-2.190 |
| D5 | 27 | 32 | 0.156 | 0.956 | 1.514 | 0.896-2.559 |
| R9 ^c^ | 7 | 164 | 3.276×10^-26^ | <10^-5^ | 0.082 | 0.011-0.611 |
| F | 6 | 143 | 9.133×10^-23^ | <10^-5^ | 0.062 | 0.027-0.142 |
| F1 | 4 | 99 | 1.111×10^-15^ | <10^-5^ | 0.064 | 0.023-0.175 |
| F1a | 2 | 71 | 3.096×10^-13^ | <10^-5^ | 0.074 | 0.027-0.203 |
| F2 | 1 | 17 | 0.006 | 0.092 | 0.102 | 0.013-0.766 |
| F3 | 1 | 12 | 0.031 | 0.418 | 0.145 | 0.019-0.118 |
| F3a | 1 | 12 | 0.031 | 0.418 | 0.145 | 0.019-1.118 |
| F4 | 0 | 7 | 0.046 | 0.553 | 0.636 | 0.610-0.662 |
| G | 26 | 31 | 0.172 | 0.962 | 1.503 | 0.882-2.564 |
| M10 | 20 | 14 | 0.009 | 0.136 | 2.580 | 1.291-5.157 |
| M12 | 2 | 8 | 0.344 | 1.000 | 0.438 | 0.093-2.069 |
| M7b | 57 | 59 | 0.003 | 0.047 | 1.795 | 1.224-2.632 |
| M7c | 27 | 32 | 0.156 | 0.946 | 1.514 | 0.896-2.559 |
| M8a | 20 | 27 | 0.359 | 0.999 | 1.317 | 0.730-2.374 |
| M9a | 10 | 11 | 0.387 | 1.000 | 1.613 | 0.680-3.826 |
| N9a | 15 | 33 | 0.563 | 1.000 | 0.793 | 0.426-1.476 |
| R11 | 2 | 5 | 1.000 | 1.000 | 0.703 | 0.136-3.636 |
| Y | 11 | 7 | 0.050 | 0.590 | 2.807 | 1.081-7.290 |
| Z | 10 | 21 | 0.641 | 1.000 | 0.835 | 0.390-1.787 |

^a^ Two tailed Fisher exact test was applied instead a Pearson chi-square test in cases containing cell counts below five

^b^ Adjusted *P*-value: adjustment of *P*-values was carried out with a permutation-based approach; number of permutations = 100,000; OR (95% CI): Odds Ratio (95% Confidence Interval)

^c^ Note that haplogroup F is a sub-haplogroup of haplogroup R9 and the number of F mtDNAs are also included here
