# Supplementary material for: Mitochondrial DNA Haplogroup Background Affects LHON, but Not Suspected LHON, in Chinese Patients
Source: PLoS One. 2011 Nov 15;6(11):e27750. doi: 10.1371/journal.pone.0027750 (PMC3216987; doi:10.1371/journal.pone.0027750)
Supplement: Table S5 — Haplogroup frequencies and Pearson's chi-square test in 479 LHON patients with m.11778G>A and 1,689 Han Chinese from the general populations. (DOC) [file pone.0027750.s006.doc]

Table S5. Haplogroup frequencies and Pearson’s chi-square test in 479 LHON patients with m.11778G>A and 1,689 Han Chinese from the general populations

| Haplogroup | LHON | Pooled Han Chinese ^a^ | *P-*value ^b^ | Adjusted *P*-value ^c^ | OR | 95% CI |
| --- | --- | --- | --- | --- | --- | --- |
| A | 16 | 92 | 0.080 | 0.772 | 0.600 | 0.349-1.030 |
| B4 | 62 | 196 | 0.472 | 1.000 | 1.133 | 0.834-1.537 |
| B5 | 30 | 94 | 0.639 | 1.000 | 1.134 | 0.742-1.733 |
| C | 21 | 51 | 0.185 | 0.972 | 1.473 | 0.877-2.474 |
| D4 | 98 | 252 | 0.005 | 0.085 | 1.467 | 1.132-1.901 |
| D5 | 27 | 88 | 0.801 | 1.000 | 1.087 | 0.697-1.694 |
| R9 ^d^ | 7 | 305 | 5.807×10^-27^ | <10^-5^ | 0.067 | 0.032-0.143 |
| F | 6 | 274 | 1.999×10^-24^ | <10^-5^ | 0.066 | 0.029-0.148 |
| F1 | 4 | 193 | 1.118×10^-14^ | <10^-5^ | 0.075 | 0.028-0.204 |
| F1a | 2 | 132 | 1.318×10^-12^ | <10^-5^ | 0.086 | 0.032-0.234 |
| F2 | 1 | 48 | 0.001 | 0.018 | 0.072 | 0.010-0.519 |
| F3 | 1 | 15 | 0.125 | 0.908 | 0.234 | 0.031-1.772 |
| F3a | 1 | 13 | 0.178 | 0.969 | 0.270 | 0.035-2.067 |
| F4 | 0 | 8 | 0.131 | 0.921 | 0.778 | 0.761-0.796 |
| G | 26 | 71 | 0.308 | 1.000 | 1.308 | 0.825-2.074 |
| M10 | 20 | 35 | 0.016 | 0.246 | 2.059 | 1.177-3.601 |
| M12 | 2 | 4 | 0.619 | 1.000 | 1.766 | 0.323-9.672 |
| M7b | 57 | 119 | 0.001 | 0.018 | 1.782 | 1.277-2.487 |
| M7c | 27 | 54 | 0.019 | 0.273 | 1.809 | 1.126-2.904 |
| M8a | 20 | 63 | 0.654 | 1.000 | 1.125 | 0.673-1.880 |
| M9a | 10 | 29 | 0.731 | 1.000 | 1.220 | 0.591-2.523 |
| N9a | 15 | 62 | 0.672 | 1.000 | 0.848 | 0.478-1.505 |
| R11 | 2 | 10 | 1.000 | 1.000 | 0.704 | 0.154-3.224 |
| Y | 11 | 16 | 0.034 | 0.453 | 2.458 | 1.133-5.332 |
| Z | 10 | 50 | 0.304 | 0.995 | 0.700 | 0.352-1.389 |

^a^ Pooled Han Chinese were from reported populations (see supplementary Table 1 for more information).

^b^ Two tailed Fisher exact test was applied instead a Pearson chi-square test in cases containing cell counts below five

^c^ Adjusted *P*-value: adjustment of *P*-values was carried out with a permutation-based approach; number of permutations = 100,000; OR (95% CI): Odds Ratio (95% Confidence Interval)

^d^ Note that haplogroup F is a sub-haplogroup of haplogroup R9 and the number of F mtDNAs are also included here
