# Supplementary material for: Mitochondrial DNA Haplogroup Background Affects LHON, but Not Suspected LHON, in Chinese Patients
Source: PLoS One. 2011 Nov 15;6(11):e27750. doi: 10.1371/journal.pone.0027750 (PMC3216987; doi:10.1371/journal.pone.0027750)
Supplement: Table S6 — Haplogroup frequencies and Pearson's chi-square test in 175 LHON patients with m.11778G>A and 843 patients with suspected LHON. (DOC) [file pone.0027750.s007.doc]

Table S6. Haplogroup frequencies and Pearson’s chi-square test in 175 LHON patients with m.11778G>A and 843 patients with suspected LHON

| Haplogroup | LHON ^a^ | Suspected LHON | *P*-value ^b^ | Adjusted *P*-value ^c^ | OR | 95% CI |
| --- | --- | --- | --- | --- | --- | --- |
| A | 10 | 31 | 0.212 | 0.992 | 1.587 | 0.763-3.301 |
| B4 | 19 | 126 | 0.159 | 0.845 | 0.693 | 0.415-1.157 |
| B5 | 9 | 54 | 0.528 | 0.100 | 0.792 | 0.384-1.636 |
| C | 7 | 35 | 0.927 | 0.100 | 0.962 | 0.420-2.202 |
| D4 | 40 | 115 | 0.002 | 0.016 | 1.876 | 1.252-2.809 |
| D5 | 14 | 32 | 0.015 | 0.053 | 2.204 | 1.150-4.223 |
| R9 ^d^ | 3 | 164 | 1.678×10^-11^ | <10^-5^ | 0.072 | 0.023-0.229 |
| F | 3 | 143 | 1.838×10^-9^ | <10^-5^ | 0.085 | 0.027-0.271 |
| F1 | 2 | 99 | 1.064×10^-6^ | <10^-5^ | 0.087 | 0.021-0.357 |
| F1a | 1 | 71 | 2.731×10^-5^ | <10^-5^ | 0.062 | 0.009-0.453 |
| F2 | 1 | 17 | 0.339 | 0.981 | 0.279 | 0.037-2.112 |
| F3 | 0 | 12 | 0.238 | 0.904 | 0.826 | 0.803-0.850 |
| F3a | 0 | 12 | 0.238 | 0.904 | 0.826 | 0.803-0.850 |
| F4 | 0 | 7 | 0.611 | 0.100 | 0.827 | 0.804-0.851 |
| G | 12 | 31 | 0.057 | 0.699 | 1.928 | 0.970-3.834 |
| M10 | 7 | 14 | 0.048 | 0.263 | 2.467 | 0.981-6.206 |
| M12 | 2 | 8 | 0.685 | 0.100 | 1.207 | 0.254-5.732 |
| M7b | 15 | 59 | 0.466 | 1.000 | 1.246 | 0.689-2.251 |
| M7c | 12 | 32 | 0.070 | 0.762 | 1.866 | 0.941-3.699 |
| M8a | 9 | 27 | 0.206 | 0.990 | 1.639 | 0.757-3.548 |
| M9a | 1 | 11 | 0.703 | 0.100 | 0.435 | 0.056-3.389 |
| N9a | 3 | 33 | 0.181 | 0.952 | 0.428 | 0.130-1.412 |
| R11 | 2 | 5 | 0.345 | 0.999 | 1.938 | 0.373-10.069 |
| Y | 6 | 7 | 0.005 | 0.092 | 4.240 | 1.407-12.775 |
| Z | 2 | 21 | 0.404 | 1.000 | 0.453 | 0.105-1.948 |

^a^ The LHON patients were from our previous study . Note that Le251 was wrongly classified in that study and was corrected as haplogroup Z in the current analysis.

^b^ Two tailed Fisher exact test was applied instead a Pearson chi-square test in cases containing cell counts below five

^c^ Adjusted *P*-value: adjustment of *P*-values was carried out with a permutation-based approach; number of permutations = 100,000; OR (95% CI): Odds Ratio (95% Confidence Interval)

^d^ Note that haplogroup F is a sub-haplogroup of haplogroup R9 and the number of F mtDNAs are also included here

**Supplementary reference**

1. Ji Y, Zhang A-M, Jia X, Zhang Y-P, Xiao X, et al. (2008) Mitochondrial DNA haplogroups M7b1'2 and M8a affect clinical expression of leber hereditary optic neuropathy in Chinese families with the m.11778G>A mutation. Am J Hum Genet 83:760-768
