# Supplementary material for: Mitochondrial DNA Haplogroup Background Affects LHON, but Not Suspected LHON, in Chinese Patients
Source: PLoS One. 2011 Nov 15;6(11):e27750. doi: 10.1371/journal.pone.0027750 (PMC3216987; doi:10.1371/journal.pone.0027750)
Supplement: Table S7 — Haplogroup frequencies and Pearson's chi-square test in 175 LHON patients with m.11778G>A and 1,689 Han Chinese from general populations. (DOC) [file pone.0027750.s008.doc]

Table S7. Haplogroup frequencies and Pearson’s chi-square test in 175 LHON patients with m.11778G>A and 1,689 Han Chinese from general populations

| Haplogroup | LHON ^a^ | Pooled Han Chinese ^b^ | *P-*value ^c^ | Adjusted *P*-value ^d^ | OR | 95% CI |
| --- | --- | --- | --- | --- | --- | --- |
| A | 10 | 92 | 0.882 | 1.000 | 1.052 | 0.537-2.060 |
| B4 | 19 | 196 | 0.768 | 1.000 | 0.928 | 0.563-1.528 |
| B5 | 9 | 94 | 0.816 | 1.000 | 0.920 | 0.456-1.857 |
| C | 7 | 51 | 0.477 | 1.000 | 1.338 | 0.598-2.996 |
| D4 | 40 | 252 | 0.006 | 0.043 | 1.690 | 1.158-2.464 |
| D5 | 14 | 88 | 0.122 | 0.470 | 1.582 | 0.880-2.845 |
| R9 ^e^ | 3 | 304 | 6.462×10^-11^ | <10^-5^ | 0.079 | 0.025-0.251 |
| F | 3 | 274 | 1.993×10^-9^ | <10^-5^ | 0.090 | 0.029-0.284 |
| F1 | 2 | 193 | 7.694×10^-7^ | <10^-5^ | 0.090 | 0.022-0.364 |
| F1a | 1 | 132 | 3.642×10^-5^ | <10^-5^ | 0.068 | 0.009-0.488 |
| F2 | 1 | 48 | 0.081 | 0.708 | 0.196 | 0.027-1.432 |
| F3 | 0 | 15 | 0.387 | 0.999 | 0.905 | 0.892-0.919 |
| F3a | 0 | 13 | 0.625 | 1.000 | 0.905 | 0.892-0.919 |
| F4 | 0 | 8 | 1.000 | 1.000 | 0.906 | 0.893-0.919 |
| G | 12 | 71 | 0.105 | 0.890 | 1.678 | 0.891-3.158 |
| M10 | 7 | 35 | 0.102 | 0.485 | 1.969 | 0.861-4.502 |
| M12 | 2 | 4 | 0.102 | 0.808 | 4.870 | 0.886-26.781 |
| M7b | 15 | 119 | 0.457 | 1.000 | 1.237 | 0.706-2.168 |
| M7c | 12 | 54 | 0.013 | 0.219 | 2.229 | 1.168-4.252 |
| M8a | 9 | 63 | 0.356 | 1.000 | 1.399 | 0.684-2.865 |
| M9a | 1 | 29 | 0.355 | 0.998 | 0.329 | 0.045-2.430 |
| N9a | 3 | 62 | 0.275 | 0.965 | 0.458 | 0.142-1.474 |
| R11 | 2 | 10 | 0.313 | 0.997 | 1.941 | 0.422-8.931 |
| Y | 6 | 16 | 0.004 | 0.059 | 3.712 | 1.434-9.613 |
| Z | 2 | 50 | 0.227 | 0.979 | 0.379 | 0.091-1.571 |

^a^ The LHON patients were from our previous study . Note that Le251 was wrongly classified in that study and was corrected as haplogroup Z in the current analysis.

^b^ Pooled Han Chinese were from reported populations (see supplementary Table 1 for more information)

^c^ Two tailed Fisher exact test was applied instead a Pearson chi-square test in cases containing cell counts below five

^d^ Adjusted *P*-value: adjustment of *P*-values was carried out with a permutation-based approach; number of permutations = 100,000; OR (95% CI): Odds Ratio (95% Confidence Interval)

^e^ Note that haplogroup F is a sub-haplogroup of haplogroup R9 and the number of F mtDNAs are also included here

**Supplementary reference**

1. Ji Y, Zhang A-M, Jia X, Zhang Y-P, Xiao X, et al. (2008) Mitochondrial DNA haplogroups M7b1'2 and M8a affect clinical expression of leber hereditary optic neuropathy in Chinese families with the m.11778G>A mutation. Am J Hum Genet 83:760-768
