# Supplementary material for: Mitochondrial DNA Haplogroup Background Affects LHON, but Not Suspected LHON, in Chinese Patients
Source: PLoS One. 2011 Nov 15;6(11):e27750. doi: 10.1371/journal.pone.0027750 (PMC3216987; doi:10.1371/journal.pone.0027750)
Supplement: Table S8 — Comparison of the non-synonymous (NS) and synonymous (S) substitutions at the terminal branch level in the phylogenetic tree between 12 LHON patients belonging to haplogroup M7b1′2 and 69 reported Chinese complete mtDNAs from the general populations. (DOC) [file pone.0027750.s009.doc]

Table S8. Comparison of the non-synonymous (NS) and synonymous (S) substitutions at the terminal branch level in the phylogenetic tree between 12 LHON patients belonging to haplogroup M7b1’2 and 69 reported Chinese complete mtDNAs from the general populations

| Gene | General population  (n=69)^a^ | | LHON patients  (n=12) | | *P* ^d^ |
| --- | --- | --- | --- | --- | --- |
|  | NS ^b^ | S ^c^ | NS ^b^ | S ^c^ |  |
| *MT-ND1* | 8 | 11 | 0 | 1 | 1.000 |
| *MT-ND2* | 4 | 6 | 0 | 4 | 0.251 |
| *MT-CO1* | 7 | 7 | 4 | 4 | 1.000 |
| *MT-CO2* | 2 | 5 | 0 | 2 | 1.000 |
| *MT-ATP8* | 1 | 4 | 0 | 0 | - |
| *MT-ATP6* | 11 | 2 | 1 | 0 | 1.000 |
| *MT-CO3* | 6 | 3 | 0 | 3 | 0.182 |
| *MT-ND3* | 1 | 1 | 3 | 2 | 1.000 |
| *MT-ND4L* | 0 | 6 | 0 | 0 | - |
| *MT-ND4* | 1 | 14 | 0 | 5 | 1.000 |
| *MT-ND5* | 7 | 19 | 2 | 0 | 0.095 |
| *MT-ND6* | 0 | 8 | 0 | 0 | - |
| *MT-CYB* | 5 | 9 | 1 | 1 | 1.000 |
| Total | 53 | 95 | 11 | 22 | 0.843 |

^a^ Data from our previous study [c.f. Ref 1 and references therein].

^b^ NS refers to the number of non-synonymous substitutions.

^c^ S refers to the number of synonymous substitutions.

^d^ *P* values were determined by the Fisher’s exact test.

**Supplementary reference**

1. Zou Y, Jia X, Zhang A-M, Wang W-Z, Li S, et al. (2010) The MT-ND1 and MT-ND5 genes are mutational hotspots for Chinese families with clinical features of LHON but lacking the three primary mutations. Biochem Biophys Res Commun 399: 179-185
