# Supplementary material for: Mitochondrial DNA Haplogroup Background Affects LHON, but Not Suspected LHON, in Chinese Patients
Source: PLoS One. 2011 Nov 15;6(11):e27750. doi: 10.1371/journal.pone.0027750 (PMC3216987; doi:10.1371/journal.pone.0027750)
Supplement: Methods S1 — Supplementary methods for power calculations. (DOC) [file pone.0027750.s010.doc]

**Methods S1. Supplementary methods for power calculations**

Power calculations were performed using the Quanto software [1], although this software was specifically designed for autosomal data; see comments in Salas et al. [2].

**Statistical power of the test**

Considering an average population minimum allele frequency (MAF) of 10%, the power to detect odds ratios as low as 1.5 for risk or protective haplogroups was above 80% in all the cases: a) 98% when considering suspected LHON patients vs*.* general Han Chinese, b) 84% when considering LHON patients vs. suspected LHON patients, and c) 91% for the comparisons between LHON patients vs. general Han Chinese.

**Supplementary references**

1. Gauderman WJ (2002) Sample size requirements for matched case-control studies of gene-environment interaction. Stat Med 21: 35-50

2. Salas A, Fachal L, Marcos-Alonso S, Vega A, Martinon-Torres F (2009) Investigating the role of mitochondrial haplogroups in genetic predisposition to meningococcal disease. PLoS ONE 4: e8347
